# Polydatin down-regulates the phosphorylation level of Creb and induces apoptosis in human breast cancer cell

**DOI:** 10.1371/journal.pone.0176501

**Published:** 2017-05-03

**Authors:** Sijia Chen, Jialong Tao, Fengyun Zhong, Yang Jiao, Jiaying Xu, Qiang Shen, Haichao Wang, Saijun Fan, Yusong Zhang

**Affiliations:** 1 Department of Oncology, The Second Affiliated Hospital of Soochow University, Suzhou, Jiangsu, P.R. China; 2 School of Radiation Medicine and Protection, Medical College of Soochow University, Suzhou, Jiangsu, P.R. China; 3 Department of Clinical Cancer Prevention, Division of Cancer Prevention and Population Sciences, The University of Texas MD Anderson Cancer Center, Houston, Texas, United States of America; 4 The Feinstein Institute for Medical Research, 350 Community Drive, Manhasset, New York, United States of America; University of South Alabama Mitchell Cancer Institute, UNITED STATES

## Abstract

Polydatin (PD), a component isolated from *Polygonum cuspidatum*, has a number of biological functions. However, the antitumor activity of PD has been poorly investigated. In this study, the effect of PD on cell proliferation was evaluated by thiazolyl blue tetrazolium bromide assay. Cell cycle distribution and apoptosis were investigated by flow cytometry. The phosphorylation levels of panel of phosphor-kinases were detected by human phospho-kinase arrays. The expression of several proteins associated with cell cycle and apoptosis were analyzed by Western blot analysis. Results showed that PD effectively inhibited the growth of MDA-MB-231 and MCF-7 breast cancer cell lines. Cell cycle analysis demonstrated that PD induced S-phase cell cycle arrest. Human phosphor-kinase arrays showed that the phosphorylation level of cAMP response element-bingding proteins(Creb) was down-regulated, and the results were further confirmed by Western blot analysis. Western blot analysis showed that the expression of protein of cyclin D1 decreased in a time- and dose- dependent manner. Results suggest that PD is a potential therapeutic natural compound.

## Introduction

Breast cancer is the most frequently diagnosed cancer and the leading cause of cancer death among females worldwide, with an estimated 1.7 million cases and 521,900 deaths in 2012. Breast cancer alone accounts for 25% of all cancer cases and 15% of all cancer death among females [1]. Advances in surgery, radiotherapy, hormonal therapy, and chemotherapy have improved the treatment outcome of breast cancer. However, more than 410,000 women still die from this disease every year [2]. To date, chemotherapy has become the most frequently used therapeutic strategy for breast cancer. Furthermore, the outcome of chemotherapy in patients with advanced breast cancer is poor, thus highlighting the need for novel chemotherapy agent.

**Fig 1:**
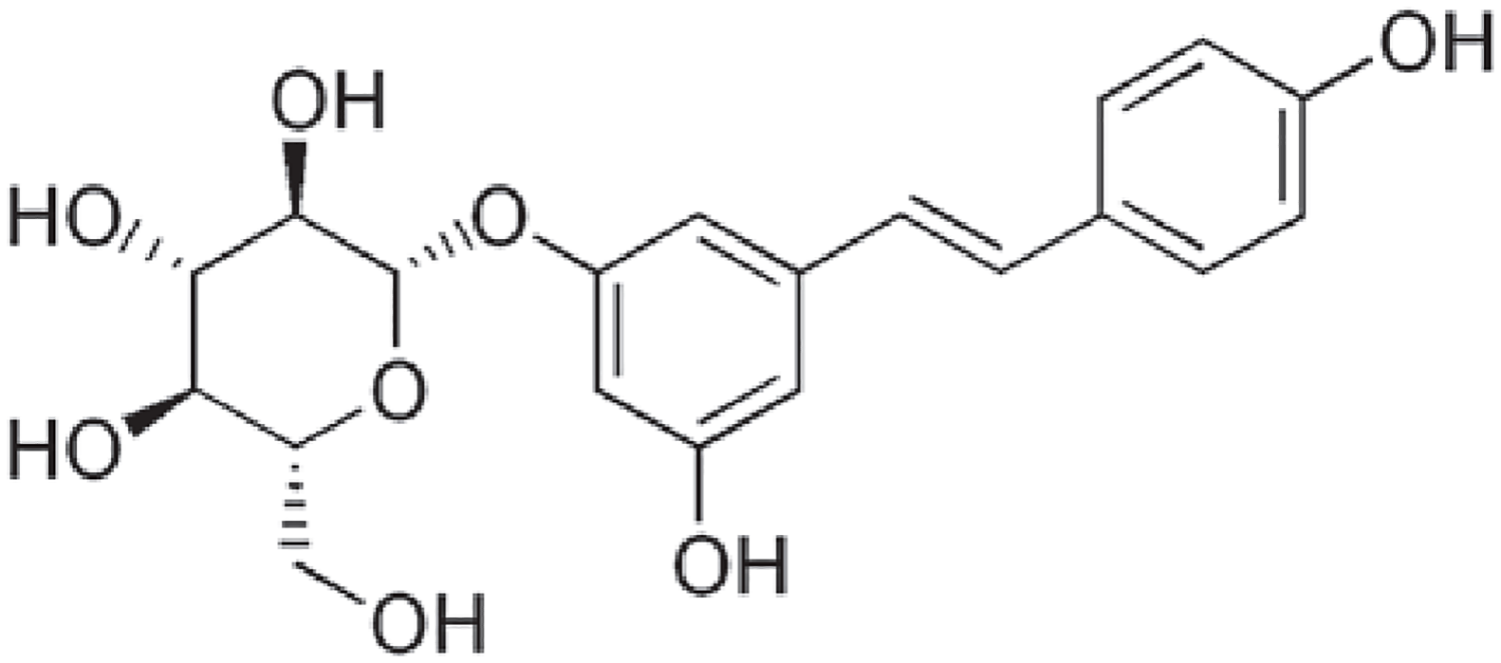
Chemical structure of polydatin.

As described previously[3], polydatin(PD) is a glycoside of resveratrolin which the glycoside group is bonded in the C-3 position and substitutes a hydroxyl group. (the chemical structure of PD is shown in Fig 1). This substitution leads to conformational changes in the molecule, thus resulting in changes in its biological properties. PD is more efficiently absorbed and more resistant to enzymatic oxidation than resveratrol and is soluble in hot water. In contrast to resveratrol, which penetrates cells passively, PD enters cells via an active mechanism using glucose carriers. These properties provide PD with greater bioavailability than resveratrol. Previous studies have demonstrated the chemo-preventive and anticancer activities of resveratrol [4–13]. However, few previous studies have analyzed the effects of PD on human cancer cells. In the currentstudy, the effects of PD on the proliferation, cell cycle phase distribution, and apoptosis of human breast cancer cell lines as well as potential underlying mechanisms, were investigated.

## Materials and methods

### Chemicals

LKT Laboratories Inc. (St Paul, MN, USA) was the supplier of the PD (Catalog No. P5845)used in this study. PD was dissolved in a stock solution of 10 mmol/l dimethysulfoxide (DMSO) and was directly diluted in amedium to appropriate concentrations prior to the experiments. Thiazolyl blue tetrazolium bromide (MTT; Catalog No. M2128) was purchased from Sigma-Aldrich (St. Louis, MO, USA). Muse Cell Cycle Kit (Catalog No. MCH100106) and Muse Annexin V & Dead Cell Kit (Catalog No. MCH100105) were obtained from Millipore Corporation (Hayward, CA, USA). Human Phospho-Kinase Array Kit (catalog Number ARY003) and Human Apoptosis Array Kit (Catalog Number ARY009) were purchased from R&D Systems Inc. (Minneapolis, MN, USA). Primary antibodies against cAMP response element-binding protein(Creb), P-Creb, and cyclin D1 and secondary antibodies were purchased from Santa Cruz Biotechnology Inc. (Santa Cruz, CA, USA). Bio-Rad Protein Assay Kit II was supplied by Bio-Rad (Hercules, CA, USA), and the enhanced chemiluminescent Western blot detection reagents (Catalog No. RPN2106) were obtained from Amersham Pharmacia Biotech (Amersham, UK).

### Cell lines and cell culture

Cancer cell lines were purchased from American Type Culture Collection (Manassas, VA, USA). The cells were maintained as a monolayer in DMEM or RPMI-1640 medium supplemented with 10% fetal calf serum, 2mM glutamine, 100μg/ml streptomycin, and 100 U/ml penicillin in a humidified atmosphere containing 5% CO_2_. Cells in the logarithmic phase were used in the experiments.

### MTT viability assay

The determination of cell viability was performed using MTT assay as described previously [14]. Cells were incubated in flat-bottom 96-well plates (6 × 10^3^ cells/well) overnight. Thereafter, cells were treated with DMSO (0.1%) or an increasing dosage of PD. Following 20, and 44 h of treatment, 20 μl MTT (5 mg/ml) was added to each well and further incubated for 4 h. Cells were then solubilized in 150 μl DMSO. The absorbance reading was obtained using a Dynatech 96-well spectrophotometer (Dynatech Laboratories, Chantilly, VA, USA). The amount of MTT dye reduction was calculated on the basis of the difference between the absorbances at 570 and 630 nm. The cell viability in treated cells was expressed as the amount of dye reduction relative to that in untreated control cells.

### Apoptosis assays and cell cycle distribution analysis

The percentage of cells that actively underwent apoptosis was analyzed using annexin V-phycoerythrin-based immunofluorescence according to the user guide of the Muse Annexin V & Dead Cell Kit. The cells were incubated in 6-well plates (2.5 × 10^5^ cells/well) overnight. The cells were then treated with DMSO or PD for 48 h. Adherent and floating cells were collected, washed in cold phosphate-buffered saline (PBS) twice, and then stained with the MuseAnnexin V & Dead Cell reagent. Apoptosis were identified using a Muse Cell Analyzer (Millipore Corporation, Hayward, CA, USA). Cells for cell cycle analysis were washed once with PBS and fixed in 70% cold ethanol for ≥4 h. The fixed cells were then washed twice with PBS, resuspended in 200μl Muse Cell Cycle Reagent, and incubated for30 min at room temperature in the dark. Cell cycle distribution was analyzed by the Muse Cell Analyzer.

### Human phosphor-kinase arrays

The phosphorylation profiles of kinases after treatment with PD were analyzed using the Human Phosphor-Kinase Array kit according to the manufacturerreatment with PD were analyzed using the Human Phosphor. incubated forusing a Muse Cell Analyzer (Millipore Corporation, Haywarf biotinylated detection antibodies, streptavidin-HRP, and chemiluminescent detection reagents were used to detect the phosphorylated cells. The relative expression of specific protein was determined by following the quantification of scanned images.

### Western blotting

Western blot assays were performed as described earlier [15]. Cells were treated with DMSO (0.1%) or PD. After 24–48h of treatment, cell lysates were prepared, and equal aliquots of protein extract were electrophoresed by SDS-PAGE. After transferring the lysates to nitrocellulose membranes, blots were blocked with 5% milk protein and were incubated using these primary antibodies for 2h or overnight: P-Creb(Catalog#: 9198,Cell Signaling 1:1000 dilution); Creb(Catalog#: 9197,Cell Signaling 1:1000 dilution); cyclinD1(Catalog#:SC-8396, Santa Cruz 1:500 dilution); cyclinA (Catalog#:SC-596, Santa Cruz 1:500 dilution); cyclinE (Catalog#:SC-247, Santa Cruz 1:1000 dilution). The blots were then reincubated with the corresponding antibodies for 1h. Blotted proteins were visualized using the enhanced chemiluminescence system, with color markers as molecular size standards. As an internal control for the amount of protein loaded, the same filter was also immunoblotted with a monoclonal β-actin(C-4, Santa Cruz 1:1000 dilution) antibody. The protein assay kit was purchased from Bio-Rad (Hercules, CA) and the enhanced chemiluminescent Western blotting detection reagents were purchased from Pharmacia Biotech (Piscataway, NJ).

### Statistical analysis

Data were expressed as mean±SD. Comparisons between the DMSO-treated control cells and PD-treated cells were made using Student’s t test. Comparisons were considered statistically significant when *P*<0.05.

## Results

### PD inhibits theproliferation ofbreast cancer cells

**Fig 2:**
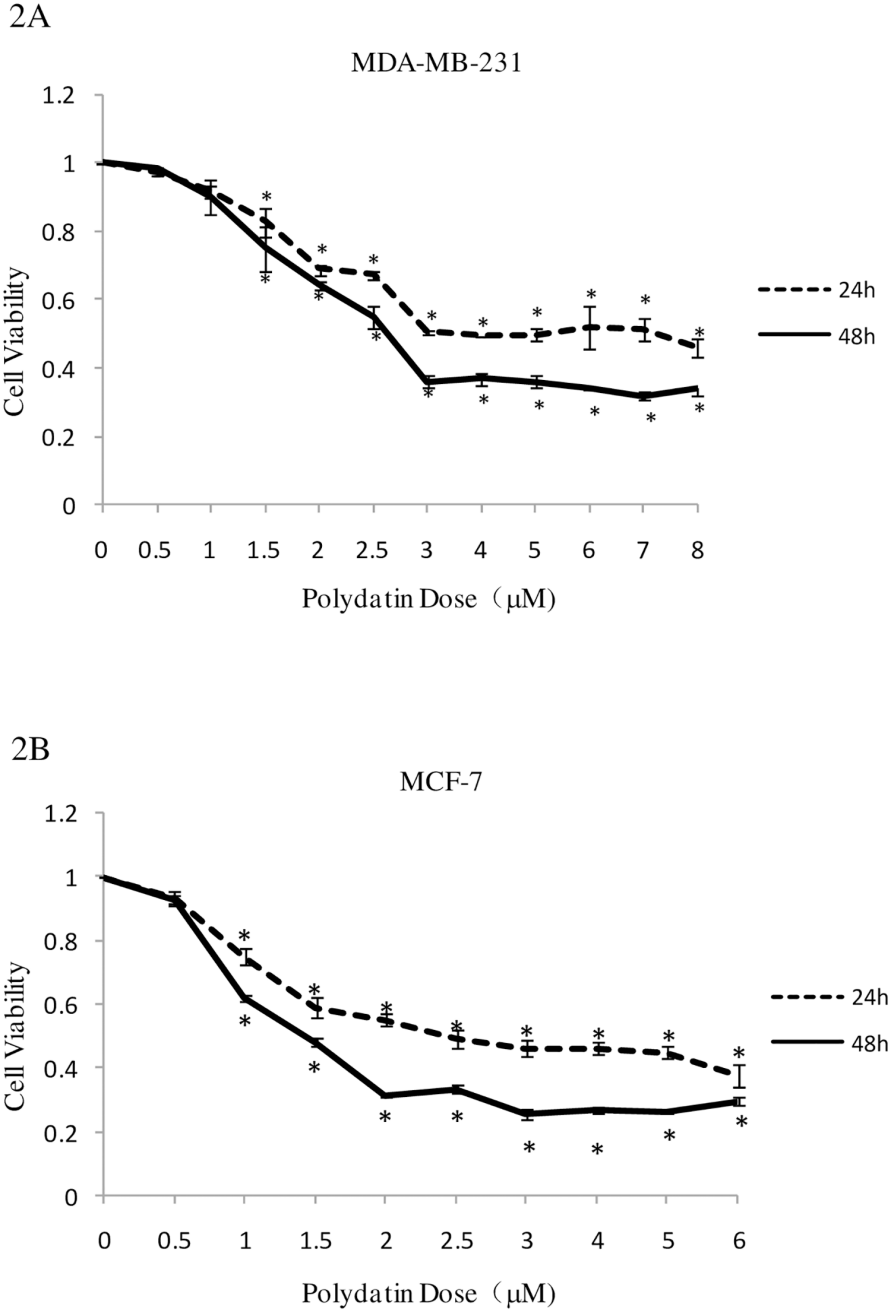
Inhibitory effects of PD on the growth of human breast cancer cells. Exponentially growing cells in 96-well plates were continuously treated with the indicated concentrations of PD for 24 and 48 h and then subjected to MTT viability assay. Dose-response curves of PD in (A) MDA-MB-231 and (B) MCF-7 cells 24 or 48 h following treatment. Data are presented as the percentage of DMSO-treated controls (mean ± SD) from three independent experiments. PD, polydatin; MTT, thiazolyl blue tetrazolium bromide; DMSO, dimethysulfoxide.

The cytotoxicity of PD on breast cancer cells MDA-MB-231 and MCF-7 was determined using MTT assay. The decrease in absorbance in this assay can be due to either a consequence of cell death or reduction in cell proliferation. As shown in Fig 2, PD inhibited the cell proliferation of breast cancer cells MDA-MB-231 and MCF-7 in a dose- and time-dependent manner.

### PD induces the S-phase cell cycle arrest of breast cancer cells

**Fig 3:**
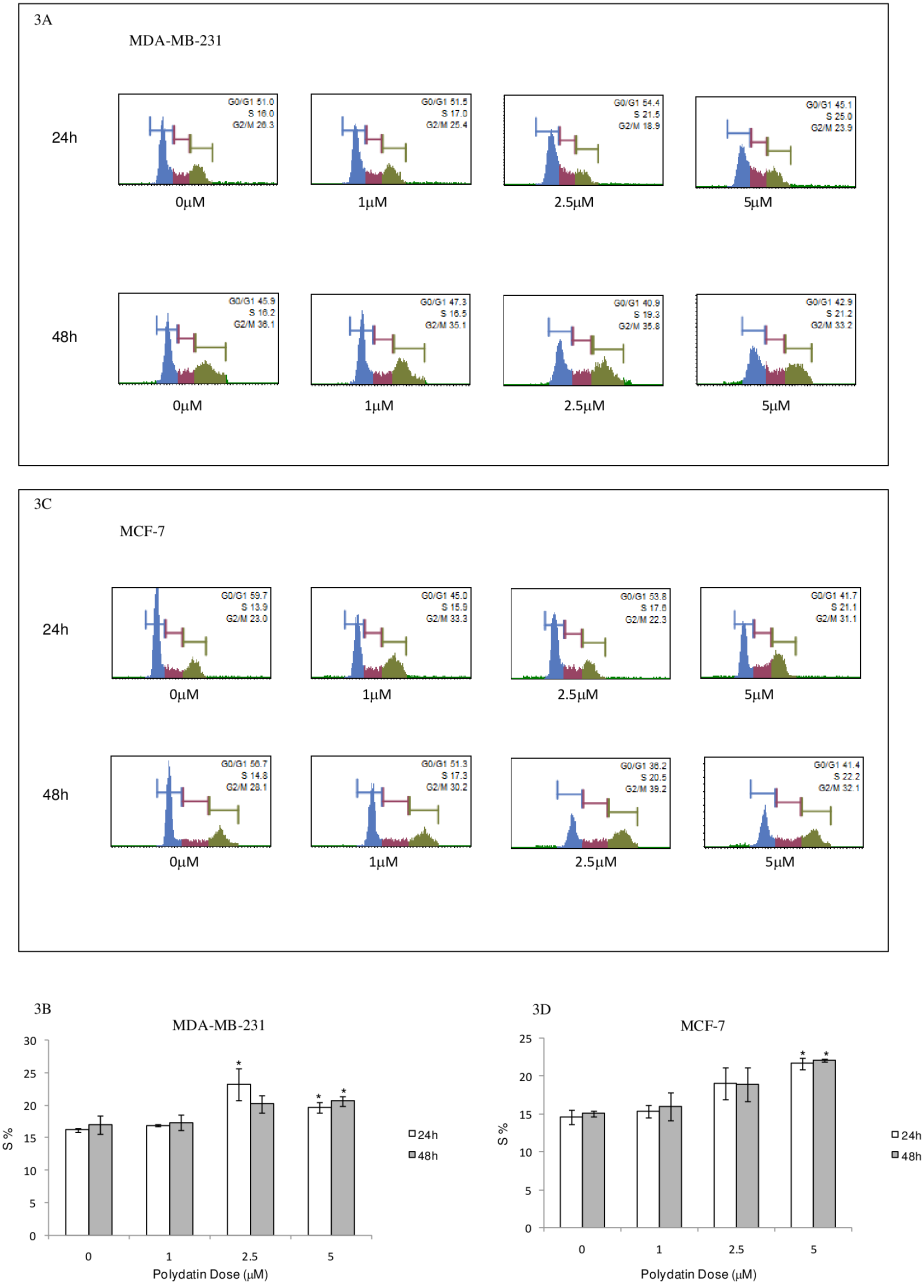
Cell cycle arrest induced by PD in human breast cancer cells. Cells were treated with increasing concentrations of PD. Following 24h and 48 h of treatment, cells were labeled with Muse Cell Cycle Reagent and then analyzed by Muse Cell Analyzer. Results indicate the percentage of cells in each phase of the cell cycle. All experiments were performed in duplicate and yielded similar results. (A) Original images of cell cycle distribution in MDA-MB-231 cells are presented. (B) The percentage of cells in S phase is presented as the mean ± SD from three various experiments in MDA-MB-231.*P<0.05, compared with the respective controls. (C) Original images of cell cycle distribution in MCF-7 cells. (D)The percentage of cells in S phase is presented as the mean ± SD from three various experiments in MCF-7 cells. *P<0.05, compared with the respective controls. PD, polydatin.

To determine the effect of PD on the cell cycle distribution of breast cancer cells MDA-MB-231 and MCF-7, we performed flow cytometry assays after MDA-MB-231 and MCF-7 cells were treated with PD for 24 and 48h. As shown in Fig 3, the treatment of cells with varying concentrations of PD for 24 and 48h resulted in the increased accumulation of cells in the S phase. PD at a concentration of 5μM(48h) increased the S-phase population from 17.01±1.41% to 20.65±0.77% in MDA-MB-231 cells, and from 15.05±3.53% to 22.05±2.12% in MCF-7 cells. These results indicate that PD can interfere with the cell cycle progression of breast cancer cells.

### PD induces the apoptosis of breast cancer cells

**Fig 4:**
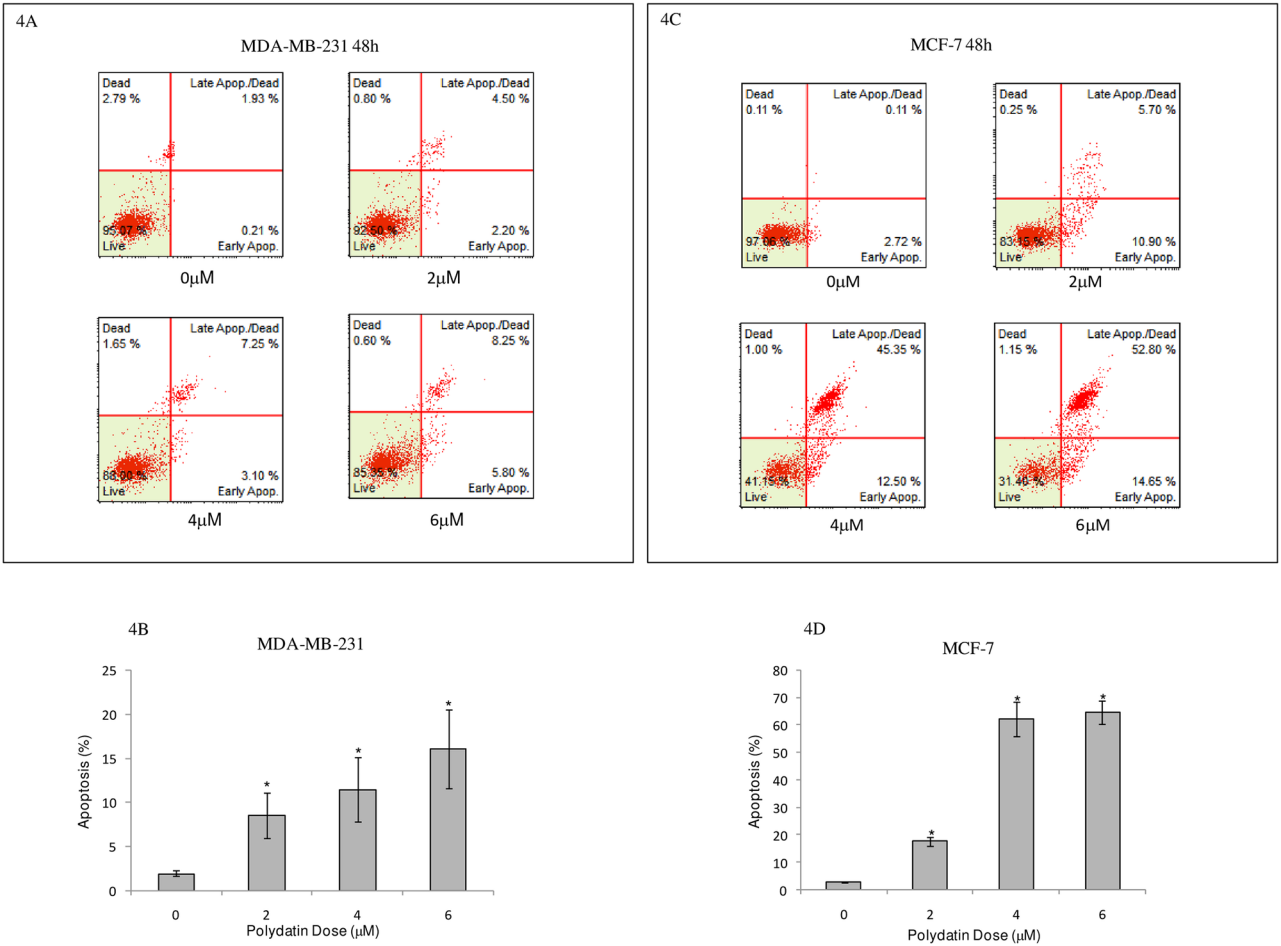
Apoptotic effects of PD on MDA-MB-231 and MCF-7 human breast cancer cells. Cells were treated with the indicated concentrations of PD for 48 h, stained with Muse Annexin V & Dead Cell reagent and then analyzed for apoptosis by Muse Cell Analyzer. The results indicate the percentage of Annexin V-positive cells (apoptosis). All experiments were performed in duplicate and yielded similar results. (A) Original images of apoptosis in MDA-MB-231 cells. (B) The percentage of cells undergoing apoptotic cell death is presented as the mean ± SD from three separate experiments in MDA-MB-231 cells. *P<0.05, vs. the respective controls. (C) Original images of apoptosis in MCF-7 cells. (D) The percentage of cells undergoing apoptotic cell death is presented as the mean ± SD from three separate experiments in MCF-7 cells. *P<0.05, compared with the respective controls. PD, polydatin.

To further evaluate the apoptosis-inducing capability of PD in breast cancer cells, breast cancer cells MDA-MB-231 and MCF-7 were treated with various concentrations of PD for 48h. Thereafter, apoptosis was detected by the Muse Cell Analyzer. As shown in Fig 4, PD induced apoptosis inMDA-MB-231 and MCF-7 breast cancer cells in a dose-dependent manner. The percentage of cells undergoing apoptotic cell death increased from 1.93±0.29% to 16.08±4.9%after exposure to 6μM of PD for 48h in MDA-MB-213 cells and from 2.75±0.11% in the control culture to 64.±5.13% after exposure to 6μM of PD for 48h in MCF-7cells.

### PD decreases the phosphorylation levels of Creb in breast cancer cells

**Fig 5:**
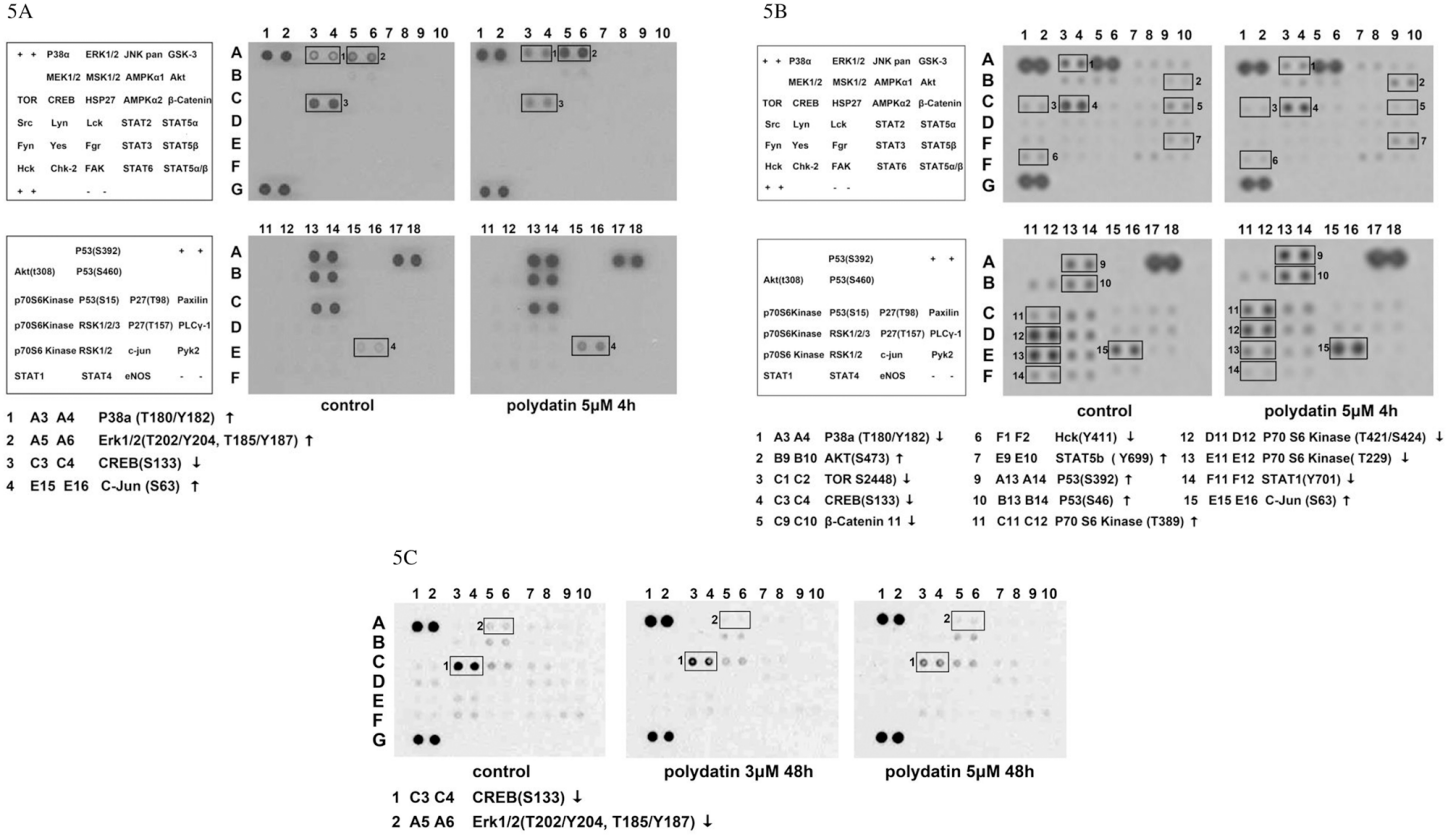
Human phosphor-kinase array analysis in response to PD treatment. Whole cell lysates were prepared from MDA-MB-231 and MCF-7 cell lines, either left untreated or exposed to PD and hybridized with a human Phosphor-Kinase array kit.(A)MDA-MB-231 cell lines were treated with PD for 4h, (B) MCF-7 cell lineswere treated with PD for 4h. (C)MDA-MB-231 cell line were treated with PD for 48h Each kinase is spotted in duplicate. The pairs of dots in each corner are positive controls. Each pair of the most positive kinase dots is denoted by a numeral, with the identity of the corresponding kinases listed as follows: Upon exposure to PD, a decrease in phosphorylated Creb was confirmed by Western blot analysis. As shown in Fig 6, the decrease in the phosphorylation levels of Creb occurred when MDA-MB-231 and MCF-7 cells were treated with PD for 2h. In MCF-7 cells, phosphorylated Crebwas still in a lower level after exposure to PD for 40h. In MDA-MB-231 cells, the phosphorylation levels of Creb began to increase after cells were treated with PD for 16h; however, the rate of increase was still lower than untreated control cells in 40h. At the same time, no obvious change was observed in the level of Creb protein.

To explore the molecular basis for the anti-proliferation activity of PD in breast cancer cells, human phosphor-kinase arrays were employed to detect the phosphorylation levels of a panel of phospho-kinases after MDA-MB-231 and MCF-7 cancer cells were treated with 5μM PD for 4 and 48h. As shown in Fig 5, the phosphorylation levels of Creb were significantly down-regulated after exposure to 5μM of PD for 4h in MDA-MB-213 cells. Simultaneously, the phosphorylation levels of P38, Erk, and Jun were up-regulated. After the MDA-MB-231 cells were treated with 3and 5μM PD for 48h, the phosphorylation levels of Creb decreased, and the degree of reduction corresponded ina dose–dependent manner. Similar results were observed in the MCF-7 cell lines.

**Fig 6:**
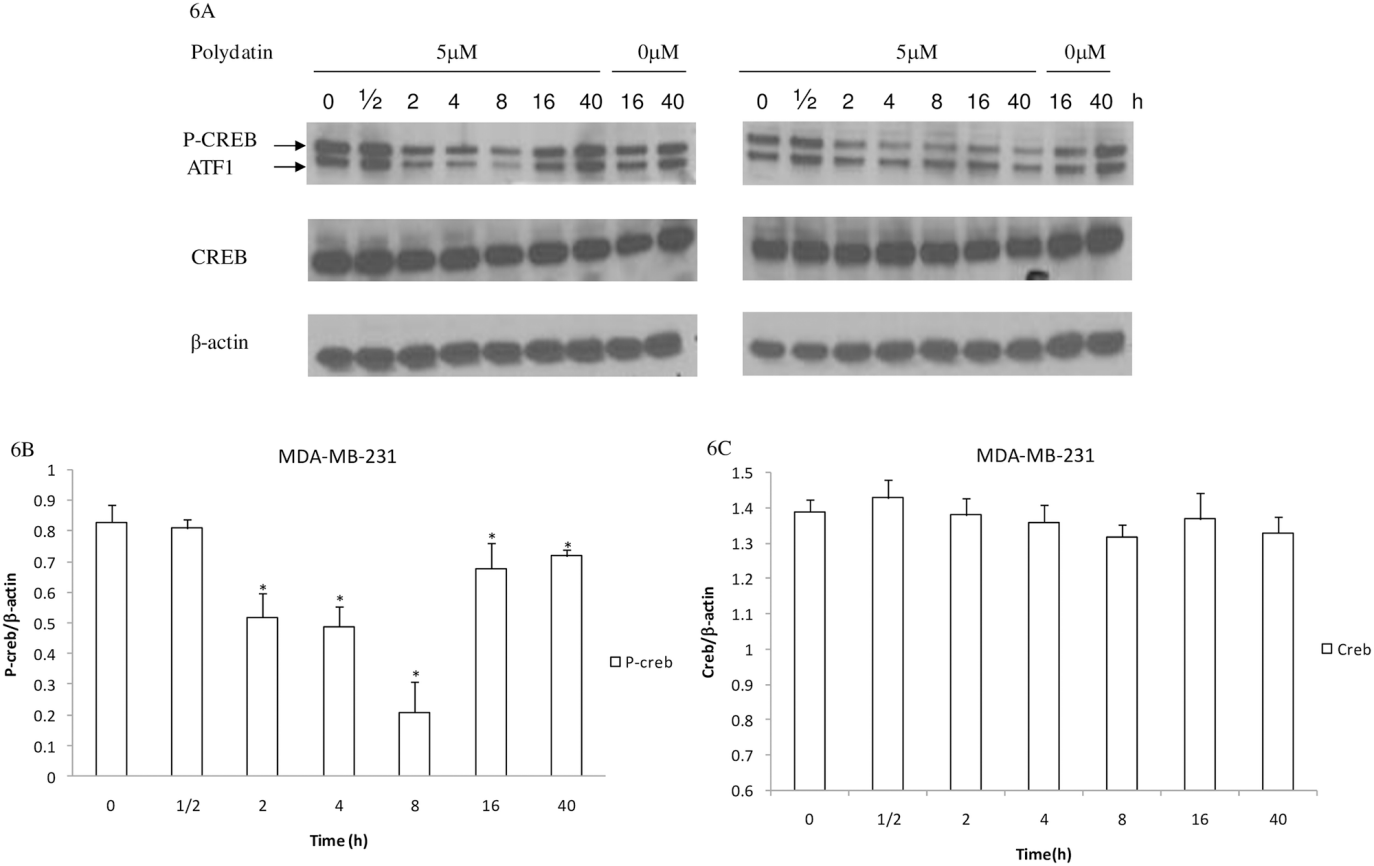
Inhibition effect of PD on the phosphorylaion levels of Creb. (A) Human breast cancer cells were treated with PD for different times. Expression levels of phospho-Creb and Creb were detected by western blot analysis and β-actin was used as a control. (B) phospho-Creb protein bands were quantitied by densitometry and the data are presented as the mean ± SD from three experiments.(C) Creb protein bands were quantitied by densitometry and the data are presented as the mean ± SD from three experiments. *P<0.05, compared with the respective controls. PD, polydatin.

### PI3K/AKT and MAPK pathways are not involved in the effect of PD on cell proliferation

**Fig 7:**
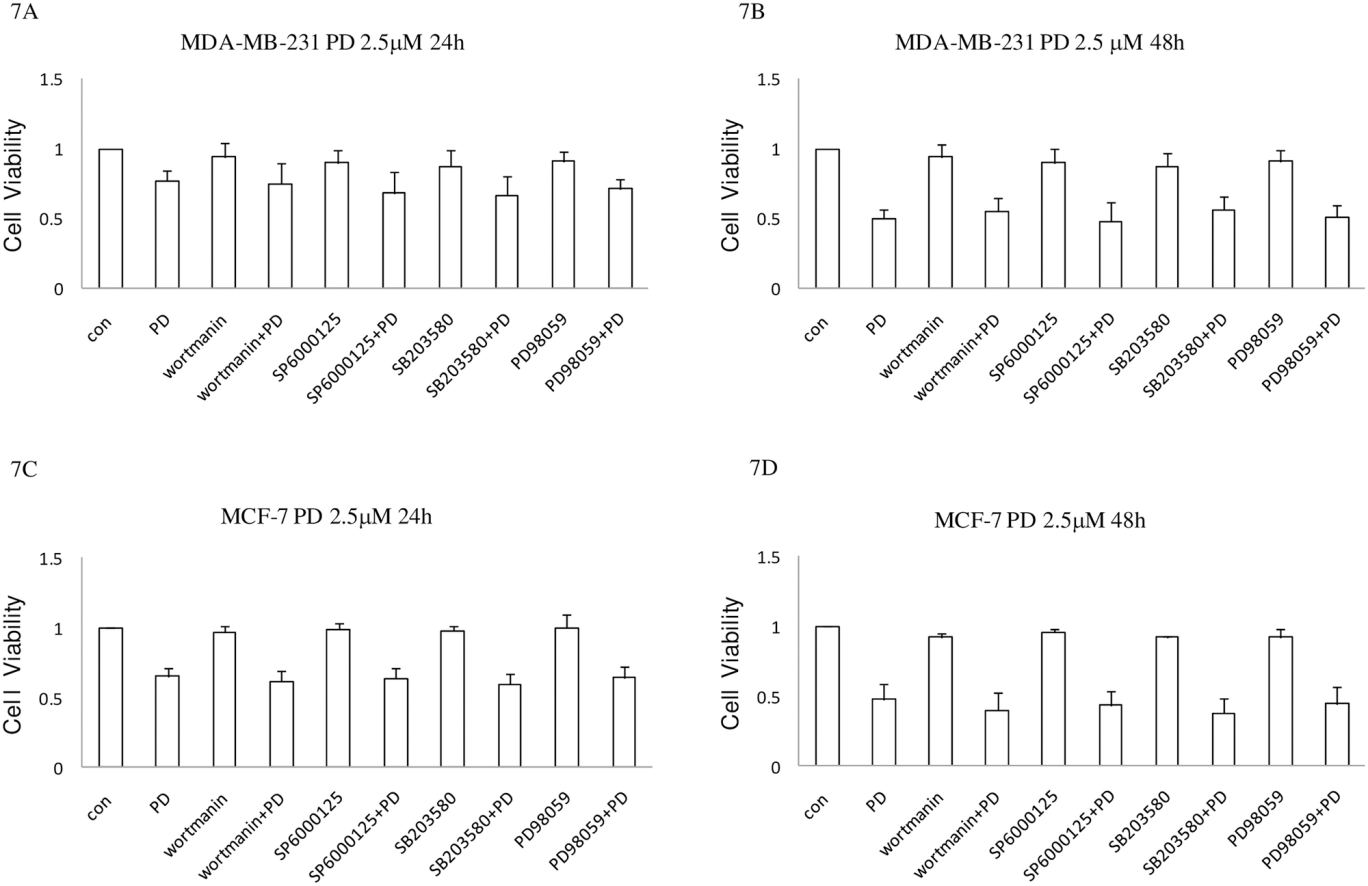
The effect of PI3K/AKT and MAPK pathways on inhibitory effects of PD in human breast cancer cells. Exponentially growing cells in 96-well plates were pre-treated with with 10μM Specific PI3K inhibitor wortmannin, ERK1/2 inhibitor PD98059, P38 inhibitor SB203580, and JNK inhibitor SP600125 for 1h. Then MDA-MB-231 and MCF-7 cells were treated by PD (2.5, μM) for 24h (A) (C) and 48h(B) (D),. And then cells were subjected to MTT viability assay. Data are presented as the percentage of DMSO-treated controls (mean ± SD) from three independent experiments. PD, polydatin; MTT, thiazolyl blue tetrazolium bromide.

The human phosphor-kinase arrays showed that the levels of Phospho-protein kinase B9(AKT), Phospho-P38, and Phospho-Erk increased in breast cancer cells after treatment by PD for 4h (Fig 5). Western Blot analysis showed that the levels of Phospho-AKT and Phospho-Erk increased first and then decreased. At the same time, no obvious change was observed in the level of the AKT and Erk proteins(datanot shown). To explore the role of phosphatidylinositol 3-kinase (PI3K)/protein kinase B9 (AKT) and mitogen-activated protein kinase (MAPK) pathways in the inhibitory effects PD on cell proliferation, breast cancer cells MDA-MB-231 and MCF-7 were pre-treated with 10μM Specific PI3K inhibitor wortmannin, ERK1/2 inhibitor PD98059, P38 inhibitor SB203580, and JNK inhibitor SP600125 for 1h. The cells were then treated by PD (2.5, μM) for 24 and 48h. As shown in Fig 7, the inhibitory effect of PD on cell proliferation was not affected by the pre-treatment of wortmannin, SB203580, SP600125, orPD98059. The cell cycle distribution and apoptosis induced by PD were not affected by wortmannin, SB203580, SP600125, or PD98059, either (data not shown).

### PD down-regulates cyclin D1 expression in breast cancer cell lines

**Fig 8:**
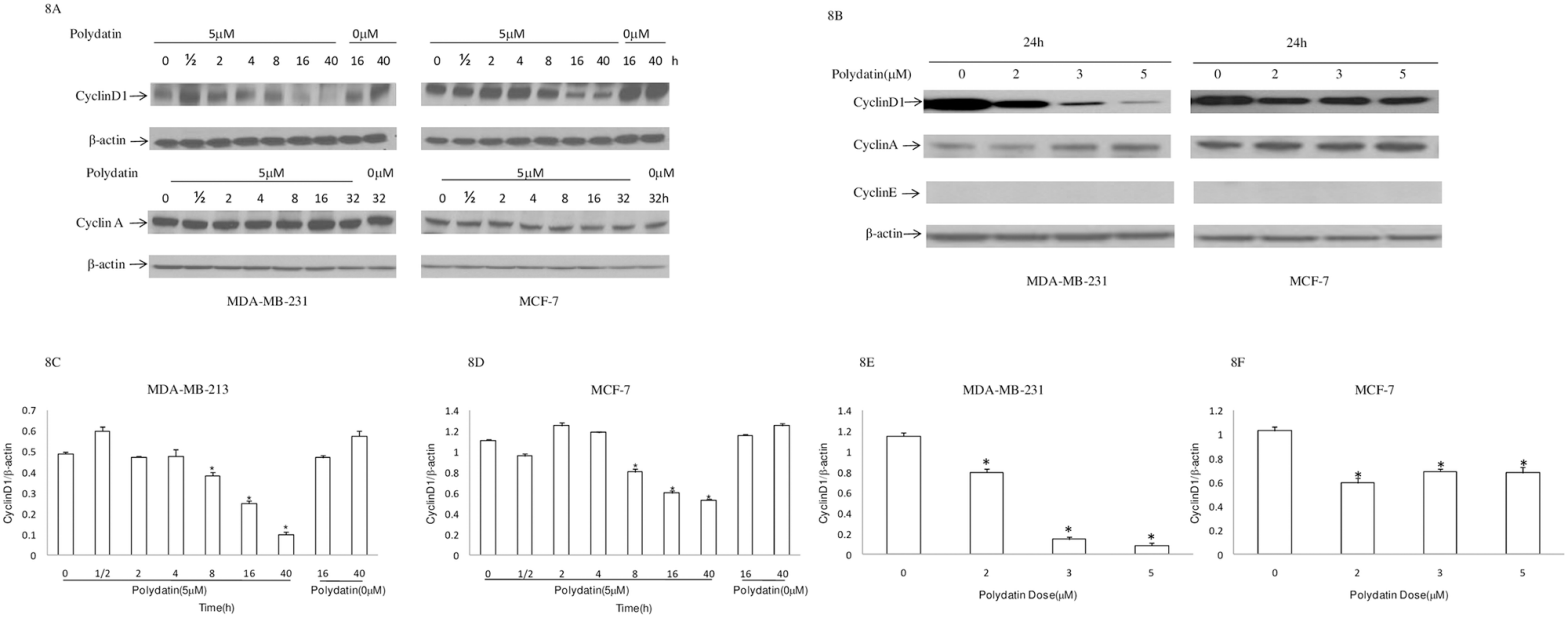
Theeffect of PD on the expression levels of cell cycle–related protein. Human breast cancer cells were treated with PD for different times(A) or different concentration(D). Expression levels of CyclinD1, CyclinA and CyclinE were detected by western blot analysis and β-actin wasused as a control. (B) (C) (E) (F) Cyclin D1 protein bands were quantitied by densitometry and the data are presented as the mean ± SD from three experiments. *P<0.05, compared with the respective controls PD, polydatin.

To explore the mechanism underlying the effects of PD on S-phase cell cycle arrest, the expression levels of cell cycle-related protein cyclin D1 were examined. The results showed (Fig 8) that the expression of protein cyclin D1 in MDA-MB-231and MCF-7 cells began to decrease following the treatment with PD for 8h, and continued after 40h of treatment. However, the expression of protein cyclin A didn’t change significantly. Furthermore, the results showed that PD induced the decrease incyclin D1 in a dose-dependent manner(Fig 8C).

## Discussion

As described previously[3], *Polygonum cuspidatum*, a traditional Chinese medicinal herb commonly used for its roots and rhizomes, has been officially listed in the Pharmacopoeia for a number of years. PD is one of the main effectivecomponents of *P*. *cuspidatum*. Previously, our study [3]demonstrated that PD could inhibit cell proliferation, induce S-phase cell cycle arrest and apoptosis in human lung cancer cells. In the present study, we evaluated the effect of PD on human breast cancer cell lines MDA-MB-231 and MCF-7, and explored the possible mechanisms. The results showed that PD could effectively inhibit the proliferation and induce the S-phase cell cycle arrest apoptosis in human breast cancer cells MDA-MB-231 and MCF-7.

Creb is a well-characterized transcription factor of the basic leucine zipper family [16]. Creb regulates a number of genes with diverse functions, including proliferation, survival, memory, and learning [17]. Recent studies showed that Creb plays an important role in the development and metastasis of different solid tumors. The level of Creb1 in breast cancer patients is elevated and is significantly up-regulated in patients with a poor prognosis, metastatic disease, and nodal involvement [18]. The phosphorylation of Ser133 and/or the nuclear translocation of the transducer of regulated Creb activity co-activators are required for Creb activation [17, 19].

In this study, we found that PD significantly decreased the phosphorylation levels of Creb in a dose-dependent manner. However, the levels of Creb protein were not affected. The results indicated that PD inhibited the activation of Creb by decreasing the phosphorylation of Creb,thus inhibiting the proliferation of breast cancer cells.

In recent studies using a genome wide analysis, Creb was found to regulate approximately 4,000 target genes. The list includes genes that are important for cell cycle control such as cyclinD1 and cyclin A [20]. CyclinD1, an important regulator of cell cycle progression, functions as a transcriptional co-regulator [21]. CyclinD1 levels must be high during the G_1_phase to initiate DNA synthesis but must be suppressed to low levels during the S phase for efficient DNA synthesis. To continue cell proliferation, cyclinD1 must be induced once again during the G_2_phase [22]. In the present study, we found that the phosphorylation levels of Creb began to decrease after breast cancer cells were treated with PD for 2h, whereas the decrease in the expression level of cyclinD1 occurred at 8h of treatment. Therefore, we speculate that the mechanism underlying the inhibitory effect of PD might be due to inhibition in Creb phosphorylation which in turn suppresses the transcription of cyclinD1, and causes cell cycle progression arrest at the S phase, eventually leading to apoptotic death of breast cancer cells.

The PI3K/AKT pathway are implicated in the regulating of proliferation, survival, and death of multiple cells. Recent studies showed that the PI3K/AKT pathway plays a key role in promoting cell proliferation and inhibiting cell apoptosis in the genesis and development of tumors, and abnormalities in this pathway were found in a variety of tumors [23–25]. In the current study, we found that the levels of Phospho-AKT increased in breast cancer cells after treatment byPD. The PI3K inhibitor wortmannin exhibited an insignificant effect on the cell survival, cell cycle, and apoptosis of PD-treated breast cancer cells. These results indicated that the PI3K pathway was not involved in the effect of PD on cell proliferation.

MAPKs include Erk(extracellular signal-regulated protein kinase), JNK(c-Jun N-terminal kinase),and P38. MAPKs are important signaling pathways in the transduction of extracellular stimuli into intracellular responses, and their functions include regulating cell proliferation, differentiation, and apoptosis [26–28]. In this study, Phospho-P38 and Phospho-Erk were increased in breast cancer cells after treatment by PD. However, cell growth inhibition, cell cycle arrest, and apoptosis induced by PD were not affected by Erk inhibitor PD98059 or p38 inhibitor SB203580. These results indicated that the MAPK pathway was not involved in the effect of PD on cell proliferation, either. The increases in levels of Phospho-AKT, Phospho-P38, and Phospho-Erk are likely due to mechanism of cell protection triggered by PD. However, this cell-protecting mechanism is not enough to counter the anti-tumor effect of PD and subsequent cell apoptosis.

In conclusion, we have performed a preliminary investigation into the inhibitory effect of PD on breast cancer cells. The anti-proliferation effect of PD involves the suppression of cell cycle progression and the induction of apoptosis in human breast cancer cells. The phosphorylation of Creb and cyclinD1 might be involved in the antitumor effect of PD. However, the antitumor effect and toxicity of PD in vivo is unknown. Future studies on the in vivo effect of PD are necessary.
